# Experiences of Perinatal Mental Health Care among Minority Ethnic Women during the COVID-19 Pandemic in London: A Qualitative Study

**DOI:** 10.3390/ijerph19041975

**Published:** 2022-02-10

**Authors:** Sabrina Pilav, Abigail Easter, Sergio A. Silverio, Kaat De Backer, Sushma Sundaresh, Sara Roberts, Louise M. Howard

**Affiliations:** 1 Oxleas NHS Foundation Trust, Bexley Bromley and Greenwich Perinatal Mental Health Service, Queen Mary’s Hospital, London DA14 6LT, UK; s.pilav@herts.ac.uk (S.P.); s.sundaresh1@nhs.net (S.S.); sara.roberts5@nhs.net (S.R.); 2 Department of Women and Children’s Health, Faculty of Life Sciences and Medicine, Kings College London, London SE1 7EH, UK; sergio.silverio@kcl.ac.uk (S.A.S.); kaat.de_backer@kcl.ac.uk (K.D.B.); 3 Section of Women’s Mental Health, Institute of Psychiatry, Psychology and Neuroscience, King’s College London, London SE5 8AF, UK; louise.howard@kcl.ac.uk

**Keywords:** COVID-19, perinatal mental health, minority ethnic women, maternity services, qualitative analysis

## Abstract

(1) Background: Approximately one in five women will experience mental health difficulties in the perinatal period. Women from ethnic minority backgrounds face a variety of barriers that can prevent or delay access to appropriate perinatal mental health care. COVID-19 pandemic restrictions created additional obstacles for this group of women. This study aims to explore minority ethnic women’s experiences of perinatal mental health services during COVID-19 in London. (2) Methods: Eighteen women from ethnic minority backgrounds were interviewed, and data were subject to a thematic analysis. (3) Results: Three main themes were identified, each with two subthemes: ‘Difficulties and Disruptions to Access’ (Access to Appointments; Pandemic Restrictions and Disruption), ‘Experiences of Remote Delivery’ (Preference for Face-to-Face Contact; Advantages of Remote Support); and ‘Psychosocial Experiences’ linked to COVID-19 (Heightened Anxiety; Social Isolation). (4) Conclusions: Women from ethnic minority backgrounds experienced disrupted perinatal mental health care and COVID-19 restrictions compounding their mental health difficulties. Services should take women’s circumstances into account and provide flexibility regarding remote delivery of care.

## 1. Introduction

Around 20% of women meet criteria for mental health diagnoses, such as depression and anxiety [1], in pregnancy or the first year postpartum (the perinatal period) [2,3]. Meta-analyses have shown that women from minority ethnic and lower socioeconomic backgrounds can be disproportionately affected by physical and psychological difficulties during the perinatal period, in comparison to white women [4]. The reasons for this are multifactored, and likely intersectional with socioeconomic status, comorbidities, language, and social complexity. Poorer access to antenatal and mental health care, reduced identification and treatment of mental illness among women from minority ethnic backgrounds, and discrimination due to race, language, and culture are also thought to play a significant role in widening health inequalities [5,6,7]. 

The global COVID-19 pandemic has been found to increase prevalence of a multitude of mental illnesses and to exacerbate pre-existing mental health difficulties, while access to mental health services was limited [8,9]. Simultaneously, research is emerging that suggests the looming threat of a global pandemic was a traumatic stressor for mental health to many, even when actual exposure to the virus was relatively contained [10]. It is, therefore, unsurprising that the global COVID-19 pandemic placed perinatal women under additional strain [11,12]. Despite this, the most highlighted concerns for women during this time have focused on the potential physical health risks of mother and baby contracting coronavirus [13,14,15,16,17,18]. There has been increasing evidence of the negative impact of the pandemic on women’s mental health and wellbeing [12,19,20,21,22,23]. However, studies have reported a lack of research focused on the experiences of women from minority ethnic backgrounds [12,21,24,25,26,27,28]. Qualitative approaches are therefore needed to capture the in-depth experiences of women who are not well represented in current research. 

During the perinatal period, it is well known that mothers may experience difficulties adjusting to their new roles and as a result often seek support from a variety of social and family networks [29]. The use of peer support networks has been found to help new mothers experiencing mental health problems feel validated, connected, and less likely to experience isolation [30]. During the pandemic, this form of support was severely lacking, leaving many reporting low mood [31], with evidence to suggest that prenatal and postnatal anxiety and depression increased during the pandemic [25,27]. As in many countries, a national lockdown and series of social restrictions were implemented across the UK in March 2020, to prevent the spread of the virus. These restrictions led to disproportionate socioeconomic difficulties being felt, alongside health care disruption and delays [32]. This disruption was also felt in maternity and perinatal mental health settings [33,34]. For maternity services, changes were implemented to restrict the presence of partners and family at various stages of pregnancy from scans to birth, with health care providers reducing face-to-face consultations on the grounds of COVID-19 transmission risks to pregnant women [26,34,35,36]. For community perinatal services, face-to-face appointments were limited, with many consultations being moved to a remote platform [37,38]. 

Women from ethnic minority backgrounds may have more difficulties accessing services and may perceive their experiences to be less favourable [39,40]. Furthermore, increased maternal age and women in more economically deprived areas may experience higher mental health risks, compared to those in less deprived areas [41]. A study into social stressors and depression rates among British Pakistani mothers found depressed mothers had more non-health related stressors such as marital, housing, and financial difficulties [42]. Given the high levels of minority ethnic employees in frontline health care roles and in the service industry, they may have disproportionately higher exposure rates to COVID-19 [43,44]. Thus, considering health inequities related to ethnicity exist on a global scale [45], and minority ethnic groups have recorded higher mortality rates, particularly during times of health emergencies [46], the risks of the pandemic on minority ethnic women pose a significant public health concern [47]. 

This research aims to explore minority ethnic women’s experiences of perinatal mental health services during the first wave of the COVID-19 pandemic in the UK, as these are currently underrepresented in perinatal mental health research. These results inform clinical recommendations to improve ethnically diverse women’s experiences of perinatal mental health care. 

## 2. Materials and Methods

### 2.1. Setting

The present study was conducted within a community perinatal mental health service in South London, covering an area of London where an estimated 17–34% of the population are from minority ethnic backgrounds [48,49,50]. The service covers three main South London boroughs: Bromley, Bexley, and Greenwich. The number of women from ethnic minority backgrounds living in these areas are set to increase [48,49,50] and across the three boroughs, 27% (Bexley), 26% (Bromley), and 61% (Greenwich) of the population is classified as being deprived [51]. Clinical guidance in the UK recommends that all women are asked about their mental health during their first antenatal appointment, and routinely throughout pregnancy and the postnatal period. Perinatal mental health services provide specialist mental health assessment and care for those identified as requiring additional support for their mental health during the perinatal period [52]. The community service offers mental health assessment, care, and treatment for women with mental health conditions who are planning a pregnancy, are pregnant, and are up to twelve months postpartum. Referrals are accepted from a range of professionals, including midwives, obstetricians, health visitors, family doctors or general practitioners (GPs), primary mental health services (commonly referred to as Improving Access to Psychological Therapy (IAPT) services), and other secondary mental health services. The service does not offer crisis or emergency care. 

### 2.2. Study Design

A qualitative approach was used to evaluate minority ethnic women’s experiences of accessing a community perinatal mental health service during the COVID-19 pandemic. Ethical approval was granted by the Research and Development Office of Oxleas NHS Foundation Trust (approval date: 11 July 2019, Datix reg: 1056). Topics covered during the interviews included women’s experiences of accessing a range of mental health, community, and maternity NHS services during the pandemic, and the impact of coronavirus on wellbeing (Appendix A). This study formed part of a wider service evaluation, aimed at investigating barriers and facilitators to accessing services among women from ethnically diverse backgrounds. Women who self-identified as being from an ethnic minority group were purposefully selected. For the purpose of this paper, we use the ethnicity of individual participants, as they self-defined it. We acknowledge this terminology may not resonate with everyone, and ethnic and cultural backgrounds are more nuanced than the broad groupings provided in this paper. However, we utilise this terminology as it is widely used and accepted by the academic and public policy communities in the UK [53]. We hope it demonstrates sensitivity around the complex issue of racial, ethnic, and cultural identities. 

### 2.3. Participants and Recruitment

Recruitment took place between May and September 2020, commencing during the UK’s first national lockdown (March–June 2020). Participants (*n* = 18) were recruited until theme saturation was established [54]. Participants were identified and recruited during regular team meetings, as agreed by clinicians who were working with them. Inclusion criteria consisted of women referred to the service who identified as ethnically diverse and were between 16 and 55 years of age. Women could participate if they were from Black, Asian, or any other minority ethnic backgrounds (including White Other). Participants represented a range of minority ethnic backgrounds, with almost half of the women who participated self-identifying as Black or Black British (*n* = 8; 44%). All participants apart from one were in the postnatal period (*n* = 17; 94%), within 12 months after the birth, and ranged in age between 19 and 46 years at time of interview (mean age = 33.4 years). Half of the participants (*n* = 9) had three or more dependents, whilst one-third were first-time mothers (*n* = 6; 33%). Women presented with a range of primary diagnoses, such as Bipolar Affective Disorder (*n* = 2; 11%), Anxiety (*n* = 3; 17%), Trauma-Related Diagnoses (including Post-Traumatic Stress Disorder (PTSD) and Emotionally Unstable Personality Disorder) (*n* = 5; 28%), Depression (including postnatal and recurrent) (*n* = 6; 33%), and Psychotic Disorders (*n* = 2; 11%). Full demographic information can be found in Table 1.

### 2.4. Data Collection

An interview schedule was devised to explore topics of access and engagement with a community perinatal mental health service, and how the pandemic might have affected this. The results provided are not limited to this and include their experiences of accessing a variety of NHS services (maternity, primary and secondary care mental health services) together with experiences of remote delivery and any other adaptations that affected their care.

Interviews were semistructured, which allowed for common questions to be asked of all participants, while allowing for flexibility in questioning style to follow-up on points made by individuals [55]. Interviews lasted 25–80 min (mean time = 45.8 min) and, subsequent to obtaining written (electronic) informed consent, were conducted via telephone using a built-in recording device. Participants were reimbursed for their time through vouchers to the value of GBP 25. One researcher conducted all the interviews (S.P.), with consultation provided throughout the interview process (A.E., S.A.S., S.R., and L.M.H.) [56]. Interviews were transcribed verbatim, and anonymised transcripts were uploaded, managed, and analysed in NVivo [57].

### 2.5. Data Analysis 

Data were analysed using a thematic analysis, which follows a six-step methodical process of dataset familiarisation, initial coding, searching for themes, reviewing themes, and finally defining and naming themes, before writing the report [58]. Inductive analysis was conducted, and codes and themes were systematically identified from the data, rather than imposing a pre-existing coding framework. Initial codes were organised into themes, and themes were refined through collapsing or merging (where groups of codes overlapped), or splitting (where groups of codes appear to differ). Codes and themes were revised iteratively in discussion with all authors to ensure accuracy and meaningfulness. Data analyses were conducted by one researcher (S.P.), with a confirmatory coding of ~20% of transcripts offered by another (K.D.B.), ensuring inter-rater reliability across codes and themes [59]. Final themes were agreed by all authors. 

## 3. Results

The majority of participants interviewed were at the postnatal stage (up to one year), with one participant interviewed during pregnancy. There were three overarching themes with two subthemes in each, summarised in Table 2: (1) Difficulties and Disruptions to Access (Access to Appointments; Pandemic Restrictions and Disruptions), (2) Experiences of Remote Delivery (Preference for Face-to-Face Contact; Advantages of Remote Support), and (3) Psychosocial Experiences Linked to COVID-19 (Heightened Anxiety; Social Isolation). 

### 3.1. Theme 1: Difficulties and Disruptions to Access

Participants were asked about their experiences of attending appointments and if the pandemic restrictions in place affected the care they received. Participants described several practical barriers to attend appointments as well as added disruptions that delayed or disrupted their care both in the hospital and in the community. The impact of delays and disruptions were felt on an emotional level, and participants described feelings of powerlessness due to decisions on care that felt out of their control. 

#### 3.1.1. Difficulties in Attending Appointments

Participants discussed a variety of challenges they faced in their life. This included finances, transport, childcare, and mental and physical health difficulties. Some participants experienced difficulties with housing, with one participant having no fixed address and others living in insecure social housing. The impact these challenges had on their engagement with the service was closely linked, with some participants describing how some of these difficulties acted as barriers. 

Participants described how they relied on charities and services to support them with finances to be able to attend appointments, and pay for household groceries and baby products.

*“Difficulties like financially and (our friend is) sometimes buying us food and veg and nappies. If she’s at home she picks up us, if not we get public transport. Sometimes charities help us with topping up oyster cards (a London public travel payment method).”*

(Participant 2, Asian Other)

They also discussed the geographical location of some hospitals or services. For some participants they described not driving and not having access to a car as a barrier to access these services.

*“Definitely if the services feel like they are really far away, I would be less likely to engage as I don’t have a car.”*

(Participant 7, Black British)

Some participants mentioned how their mental health can affect their ability to remember and therefore attend appointments.

*“Mental health, I just can’t ever remember anything so I’m too stressed so I’m thinking about something else. Or I just forgot about it… cos I’m preoccupied worrying about something else.”*

(Participant 8, Mixed Ethnicity)

#### 3.1.2. Pandemic Restrictions and Disruption

Although restrictions were in place in maternity wards for birth partners, participants described the importance of support during birth. Participants discussed changing their birthing plans as a way to try to regain control of their care at a time when delivery may have felt uncertain or anxiety-provoking due to additional risks. They described the impact restrictions in maternity wards had on their mental state, particularly during birth at times when they needed the most support. One participant discussed the importance of having their partner there as being alone would have been emotionally intolerable.

*“There were certain rules about when you could have your partner there during the labour and thereafter. And I understand it, but having had the experience I had…if I had to have gone through it without having had (my partner) there, I’m not sure I would have survived it, in the sense that I would have been absolutely certain that would have harmed myself.”*

(Participant 13, Arab)

Alongside restrictions in hospitals, participants also discussed how this affected their postnatal care. They spoke of the disruption from perinatal services as well as community midwifery and health visitors. There seemed to be a sense of feeling restricted to go into the hospital because of the risk of cases while professionals were also advised against visiting client’s homes, leaving women in a state of flux.

*“With the coronavirus it was kind of hard, because you don’t get to see them and they won’t come to your house.”*

(Participant 6, Black British)

Additionally, participants described the dangerous consequences that disruptions to care had on their physical health.

*“As a result of corona, not having face to face midwife appts, I developed pre-eclampsia really badly, and then ended up having to have my son six weeks earlier, I was really unwell and in hospital for three weeks, it was life threatening at that point. It was because I didn’t have face to face midwives, no urine test, bloods test, it was me that insisted on going in this particular day because I was having a lot of pain under my rib area and that pain related to, the midwife and the doctor said numerous time that related to the head and no one was able to check it was actually my liver failing.”*

(Participant 5, Asian)

### 3.2. Theme 2: Experiences of Remote Delivery

Participants described their experiences of receiving care both before and during the pandemic. They perceived a mixture of advantages and disadvantages to remote contact, with an overall preference for the availability of remote contact when face-to-face contact is less necessary or more difficult to access.

#### 3.2.1. Preference for Face-to-Face Contact

Contact with professionals was perceived to be less personal when this was moved to a remote platform. Participants also expressed a preference for face-to-face contact when meeting professionals for the first time, with a move to remote contact being acceptable after this point. Further to this, some participants explained that they preferred to have psychological therapy out of the home. This may have been due to distressing content being discussed in sessions and how this may be affected by being in their home context. 

One participant described being in the middle of trauma therapy (Eye Movement Desensitisation and Reprocessing—EDMR) when the first national lockdown occurred and had the experience of this being disrupted; it could not be conducted remotely as the service was still adjusting to the changes in remote working. This was described as particularly difficult as it was so abrupt and meant her entire treatment plan was subsequently altered.

*“I was having EMDR sessions with the psychologist and obviously you can’t do them over the telephone.”*

(Participant 13, Arab)

Participants also discussed the logistical issues of video calls and finding difficulty engaging with someone virtually.

*“So, I’ve got the phone contact and the video calls, so I’ve got the regular contact but it’s not the same as in person. Sometimes I find it a little bit harder, sometimes the internet has been a bit weird if you’re on a video call and it’s buffering.”*

(Participant 10, White Other)

In some cases, moving group therapy to an online platform was seen as anxiety-provoking and participants discussed how it made them less likely to engage.

*“I haven’t attended any groups because I’m not confident to do it over the phone. It would have been easier for me in person but because of COVID, I told my nurse that I’m not confident doing these calls with other people over the phone.”*

(Participant 16, White Other)

#### 3.2.2. Advantages of Remote Support

Remote support was seen as giving women more options, particularly for those who found travelling to appointments difficult. Women discussed how they found this most helpful when attending routine appointments as the convenience factor made them more likely to engage. For some women, the anonymity of audio-only appointments was described as being a facilitator for them during therapy, as they felt more comfortable in making certain disclosures that they would have found more difficult to discuss in person. Some participants also expressed they preferred use of video as a remote option when face-to-face was not available; however, most participants also explained if contact was going to be remote, audio contact was the easier option.

Participants discussed the ease of accessing appointments remotely. They discussed this would be particularly helpful when they had general follow-up or check-in appointments that did not require a full assessment of risk or needs.

*“I think sometimes the telephone thing, with this whole coronavirus I think sometimes we don’t need to go into the office for everything, I think sometimes we could just do a telephone. Sometimes obviously they do need to see the patients if they’re in a different stage or concerned with self-harm they do need somebody face to face to assess but the ones that are okay and not too bad, I think they …it could just be a telephone yeah. It could be a zoom.”*

(Participant 3, Black British)

One participant described the anonymity of remote contact being a facilitator in enabling her to share her experience as she experienced less shame and concern about being judged.

*“(…) I found it easy to speak to her (the psychologist) over the phone about some of the things like my deeper and darker things that I… feel a bit ashamed about or kind of wouldn’t really talk to anyone about. And being able to do that on the phone meant that I could almost take the paranoia of someone staring at you out of it. I know this, but it’s a bit more, freedom (to) speak…freely without feeling judged I suppose.”*

(Participant 1, Black British)

### 3.3. Theme 3: Psychosocial Experiences Linked to COVID-19

Participants described the fears associated with the pandemic and the added strain this placed on their mental health. For some participants who had exceptionally difficult trauma backgrounds from experiencing trafficking and torture, this meant experiencing triggers for their anxiety and trauma and the added impact of the lack of social support as strategies to cope with these symptoms. There was a consensus among participants that the pandemic had affected most areas of life as many of the support systems and coping strategies women would utilise in difficult circumstances were no longer there. 

#### 3.3.1. Heightened Anxiety

There was a sense of heightened anxiety, above existing mental health problems, which came from both a fear of catching the virus as a pregnant woman, and the same fears for their family’s health. Participants who were reluctant to engage with services prior to the pandemic discussed being further affected by the additional barrier to accessing services brought about by the COVID-19 restrictions.

*“It will probably make a lot more people afraid to go and see someone they don’t know now more than ever. It was hard before, but it is going to be harder now. Pregnant women have got this fear and fear is very hard to get rid of. Anyone that is pregnant or about to be pregnant has this enormous amount of fear due to coronavirus and that does need to be addressed.”*

(Participant 17, Black Other)

Similarly, this sense of heightened anxiety was recalled by participants who had partners and family working on the frontline in the National Health Service (NHS). There was a collective anxiety for fear of their family and themselves catching coronavirus and how overwhelming this was for their mental health. For some participants, this meant utilising all their mental health support strategies to manage their heightened anxiety and delaying any planned changes to psychotropic medication for fear that they could relapse due to the unprecedented stress felt at the time.

*“Because my husband is at the [hospital], as a doctor there, there was a time when he was working in ICU. That was a really, really stressful time because, I mean, I was just like… are you going to get COVID? Are we all going to get COVID, or this or that? And that was the time, probably, where I had to be really practicing everything I have. And I said I’m going to postpone the weaning off the tablets and I’m going to really be strong in practicing all that I have learned and all the support.”*

(Participant 9, Arab)

#### 3.3.2. Social Isolation 

Participants described feelings of isolation, as throughout the perinatal period they were unable to share their pregnancy experiences with family or friends in the same way. This was sometimes further compounded by other difficulties experienced in the pandemic such as employment losses and financial strains. One participant described how her housing situation was difficult and cramped, which was made worse when lockdown started.

*“But to not have that at all, and obviously during COVID you were just so isolated from friends, family, it was not a great time at all. And also my job went because of COVID, so all my stability had gone. At the time we had just moved, we were stuck in a flat, in between houses, all my stability gone.”*

(Participant 5, Asian)

Participants spoke of the difficulties of community and children’s centres closing. This added to their feelings of isolation as they were not as able to meet and mix with new mothers, losing the psychosocial support these centres facilitated.

*“It has stopped me going to the children’s centre. I still was able to keep in contact with people from the perinatal mental health team and that hasn’t been a problem, but accessing more support via the children’s centre, doing things with [friends], having a bit of structured play and having other mums and more nursery nurses to talk to, I have missed that a lot.”*

(Participant 7, Black British)

## 4. Discussion

The findings from this study emphasise the pandemic’s effect on social isolation and disrupted health care on the mental health and engagement with perinatal mental health services among women from ethnic minority backgrounds. Participants discussed preferences for flexible adaptations that were made as a result of the pandemic, i.e., a mixture of face-to-face (particularly for first appointments) and remote appointments (with some preferring telephone and others video contact), and stressed the importance of flexibility, understanding women’s individual needs and social circumstances. In positive reports of remote delivery, this meant that participants felt more connected, being able to reach clinicians remotely more often, and feeling more comfortable being in their own environment. Some participants also reported they felt more able to be open and honest about difficult feelings via telephone contact as this felt less exposing and shameful if they had to express these disclosures if the contact was in person. In some circumstances such as first appointments and for some psychological therapies it was felt that having contact remotely was less meaningful and made participants feel less able to engage and establish a trusting rapport with the clinician. 

These points were further reinforced by participants’ difficult experiences of attending appointments when they were outside the home. Many participants described difficulties including childcare issues, services being hard to access by public transport, and financial strains as the most common issues. It is well established that greater exposure to social and economic vulnerability are associated with poorer mental and physical health, and reduced access to health care particularly among migrant, refugee, and asylum-seeking populations [60]. In the perinatal population, direct associations between social and economic adversity and maternal and infant health outcomes are evident, and there is consistent evidence of differential access to perinatal mental health care [61]. Addressing social determinants of perinatal mental health such as poverty, poor housing, racism, gender-based violence, and other structural inequalities, with gender-based violence and poor housing at a universal level, is critical to improving maternal mental health [1]. 

In addition, postnatal physical and mental health care was disrupted as a result of social distancing in the community and restrictions in hospitals. They described mother’s groups being cancelled, last minute changes and adjustments to interventions and appointments. Restrictions in hospitals were confusing and described as difficult to tolerate emotionally for many women. Some described having to go through parts of the birthing experience alone and how devastating that felt, especially in the context of the pandemic. In line with previous research findings, women described a sense of heightened anxiety during the pandemic, felt throughout the perinatal period, which was said to add to additional worries and barriers about accessing services. Further, many participants described the additional mental health effects of isolation from their support networks during COVID-19. This isolation had a further detrimental impact as a result of children’s centres closing and participants describing missing this support which they were hoping to acquire from interacting with other mothers.

The COVID-19 pandemic has had a significant effect on delivery of all services for pregnant and postnatal women, from primary and secondary care, in mental health services and maternity care. Women have felt the negative impact of disruptions to maternity care, with many feeling their quality of care was affected by the restrictions that were implemented at different stages in the perinatal timeframe. Evidence suggests these restrictions may have particularly affected some minority ethnic women by not having a partner or friend there to properly communicate their concerns and wishes. Considering these women are more at risk of poorer physical health outcomes [4], not having additional support from family or partners on wards postnatally due to restrictions may have added further difficulty for them [62].

Despite there being some evidence to suggest that the number of obstetric face to face visits did not increase COVID-19 infection [63], many services implemented remote forms of contact [33,35]. As our findings showed, women’s perceptions of this care received a mixed response. On one hand it made accessing appointments easier, while on the other provided some interpersonal and logistical challenges. As demonstrated in our results, many women also experienced technical issues in virtual contact and the quality provided. If remote services should be provided in future, investment in these is needed in order to ensure nursing contact and other care is not disrupted by potential technological difficulties and women without access are not affected by digital exclusion [25]. However, some evidence suggests women feel reluctant to discuss their mental health virtually [26], and in some cases the introduction of remote appointments as standard may have added further to the social isolation felt by women [11], with this isolation being experienced differently across the socioeconomic scale [64]. Services and nursing professionals should take account of these additional difficulties women experienced during the pandemic, particularly when recent reviews have found evidence for links between social isolation and loneliness on mortality and depression [65]. This evidence is particularly concerning for some ethnically diverse women who are already at risk of higher maternal mortality and poorer mental health outcomes [4,44,62,66]. Our study findings therefore echo recent recommendations around reconfiguration of psychiatric care in the aftermath of the COVID-19 pandemic, which should prioritise those disproportionally affected by the pandemic, including perinatal women of ethnic minority backgrounds [67,68]. As highlighted by the themes of our study, careful consideration should be given to tailoring mental health care around women’s individual needs and circumstances, while balancing the resources within mental health care services.

### Strengths, Limitations, and Future Directions

To our knowledge, this is the first UK study to look at ethnically diverse women’s experiences of the COVID-19 pandemic during the perinatal period. Participants interviewed had complex social and personal circumstances (including experience of torture and trafficking), which in themselves can be additional barriers for participation in services and indeed research. A further strength is the use of qualitative methodology with semi-structured interviews, allowing women to speak freely without being restricted to preassigned ideas concerning their beliefs and feelings about the topics discussed [69]. The nature of the thematic analysis also allowed for this and for participants’ ideas to be presented in the most accurate and concise way [70]. However, due to the lockdown that was imposed in the UK during the time of recruiting for participants, interviews were conducted remotely. This meant that for some participants it may have been more challenging to establish a rapport via telephone and this may have affected the depth of discussion. Although we sought to include women from across the perinatal period, only one who was pregnant took part and the findings are therefore more reflective of women accessing perinatal mental health care postnatally. The sample size for our study was consistent with the current literature on qualitative research methodology, i.e., recruiting participants until data saturation occurs [71,72,73]. As such, this study provides an in-depth qualitative understanding of a diverse group of women in South London. Our findings do not aim to be representative of all women’s experiences and may not be more widely generalisable or representative of the experiences of women in other settings during the COVID-19 pandemic, e.g., rural services. 

## 5. Conclusions

These findings further highlight the stark impact of social isolation, uncertainty, and a perceived lack of control during the pandemic, and the compounded effect of the social restrictions and changes to service provision on women’s mental health. It is vital to identify opportunities to help reduce this impact through transparent communication, evidence-based interventions which seek to reduce stress and enhance coping [74,75], and novel approaches such as outdoor “walk-and-talk” therapy. The importance of taking women’s individual circumstances into account when they enter services was echoed across the three themes. This is most important for nursing professionals in services where they are the first point of contact for assessment. This means that perception of the service and other professionals is often formed at this stage and can be crucial in establishing engagement, determining the likelihood of whether the woman will attend future appointments. 

For minority ethnic women, this may mean understanding socioeconomic constraints and structural inequalities such as insecure immigration status [66] along with cultural and geographical aspects that may prevent or discourage them from engaging with services [11,62]. Nurses, alongside other health care professionals need to take these considerations into account when planning and adapting the care they provide for these women [76,77,78], as evidence suggests mental health services adapted for individuals from ethnically diverse backgrounds improves both engagement and mental health outcomes [79]. 

## Figures and Tables

**Table 1:** Participant demographics.

Characteristic	*n* (%)
Ethnicity:	
Black/Black British	8 (44.4%)
Asian/Asian British	4 (22.2%)
Arab	2 (11.1%)
Mixed Other	2 (11.1%)
White Other	2 (11.1%)
Age: (Mean = 33.4)	
≤25	2 (11.1%)
26–30	3 (16.6%)
31–35	5 (27.7%)
36–41	7 (38.8%)
42–46	1 (5.5%)
Number of dependents:	
1 child	6 (33.3%)
2 children	3 (16.6%)
3 children	7 (38.8%)
≥4 children	2 (11.1%)
Primary diagnosis:	
Bipolar Affective Disorder	2 (11.1%)
Anxiety	3 (16.6%)
Trauma-Related Diagnoses (incl. PTSD and EUPD)	5 (27.7%)
Depression (including postnatal and recurrent)	6 (33.3%)
Schizophrenia type disorders	2 (11.1%)
Perinatal status:	
Pregnant	1 (5.5%)
Postnatal	17 (94.4%)

**Table 2:** Summary of themes.

Overarching Theme	Subthemes
1. Difficulties and Disruptions to Access	Access to Appointments Pandemic Restrictions and Disruption
2. Experiences of Remote Delivery	Preference for Face-to-Face Contact Advantages of Remote Support
3. Psychosocial Experiences Linked to COVID-19	Heightened Anxiety Social Isolation

## Data Availability

The datasets generated and/or analysed during this study are not publicly available due to the sensitive nature of the interviews. Summary data may be shared from the corresponding author upon reasonable request, when compliant with ethical regulation of this study.

## Appendix A. Qualitative Discussion Guide (Extract)

Firstly, I would like to thank you for taking part in this service evaluation. As this is a new service and we are always looking at ways we can improve, we would like to find out about your experiences in accessing XXX services. We would like to find out about the experiences you had during or after your pregnancy. We are interested in individual coping skills and the care individuals have received. It doesn’t matter if you have or haven’t accessed services, we are just interested in your experiences.

We are keen to hear your views and there are no right or wrong answers. Your answers will be anonymised and will not affect the care you are receiving from the services. The interview typically lasts around 45 min.

*Demographics questions*

What ethnicity do you identify as?

*COVID-19 Impact*

How has coronavirus impacted your ability to contact or engage in services? (our service but also others—midwife appointments etc.)

Did you attend 1:1 therapy or groups before—have these been affected?

Have you had any telephone assessments/scheduled appointments over the phone? If YES—how have you found them compared to face-to-face contact?

If YES, were you offered an alternative? Would you have liked to have been offered remote telephone/video appointments?

Do you have any suggestions for what would help you access support remotely?

*Facilitators to access*

What advice would you have for us to improve the service or access to the service?

What care/services would you like to see us provide?

What advice would you have for women in a similar situation to you?

*Interview closing remarks*

I’ve now come to the end of my questions. Thank you for sharing your experiences with us.

Do you have anything else you’d like to add that I haven’t asked you about?

Do you have any questions for me?

(Ask if they’d like to be contacted for future research.)
